# Molecular Profiling of Druggable Targets in Clear Cell Renal Cell Carcinoma Through Targeted RNA Sequencing

**DOI:** 10.3389/fonc.2019.00117

**Published:** 2019-03-01

**Authors:** Corina N. A. M. van den Heuvel, Anne van Ewijk, Carolien Zeelen, Tessa de Bitter, Martijn Huynen, Peter Mulders, Egbert Oosterwijk, William P. J. Leenders

**Affiliations:** ^1^Department of Biochemistry, Radboud Institute for Molecular Life Sciences, Nijmegen, Netherlands; ^2^Department of Pathology, Radboud University Medical Centre, Nijmegen, Netherlands; ^3^Center for Molecular and Biomolecular Informatics, Radboud Institute for Molecular Life Sciences, Nijmegen, Netherlands; ^4^Department of Urology, Radboud University Medical Centre, Nijmegen, Netherlands

**Keywords:** cancer, renal cell carcinoma, diagnostics, prognostics, precision therapy, RNA-sequencing, single molecule molecular inversion probes

## Abstract

Clear cell renal cell carcinoma (ccRCC) comprises more than 80% of all renal cancers and when metastasized leads to a 5-year survival rate of only 10%. The high rate of therapy failure and resistance development calls for reliable methods that provide information on the actionable biological pathways and predict optimal treatment protocols for individual patients. We here applied targeted RNA sequencing (t/RNA-NGS) using single molecule Molecular Inversion Probes on tumor nephrectomy samples of five ccRCC patients, comparing tumor with healthy kidney tissues. Transcriptome profiling focused on expression of genes with involvement in ccRCC biology that can be targeted with clinically available drugs. Results confirm high expression of vascular endothelial growth factor-A (VEGF-A) in tumor tissue relative to healthy-appearing kidney, in line with the angiogenic nature of ccRCC. PDGFRα and KIT, targets of the multi-kinase inhibitor sunitinib which is one of the current choices of first-line drug in metastasized ccRCC patients, were expressed at relatively low levels in tumor tissues, whereas significantly increased in normal kidney. Of all measured druggable tyrosine kinases, MET, AXL, or EGFR were expressed at higher levels in tumors than in normal kidney tissues, although intertumor differences were observed. Using cancer cell lines we show that t/RNA-NGS gene expression profiles can be used to predict *in vitro* sensitivity to targeted drugs. In conclusion, t/RNA-NGS analysis may provide insights into the (druggable) molecular make-up of individual renal cancers, and may guide personalized therapy of renal cell cancers.

## Introduction

Clear cell renal cell carcinoma (ccRCC) is the most common type of renal cell carcinoma, comprising more than 80% of all renal cancers (1). Upon first diagnosis, 30–40% of patients have disseminated disease (2). Patients with metastasized ccRCC (m-ccRCC) respond poorly to chemotherapy or radiotherapy. While the prognosis for these patients has improved with the introduction of targeted therapies, side-effects and intrinsic or acquired resistance still lead to a 5-year survival rate of only 10% (3).

In more than 80% of ccRCCs the *von Hippel-Lindau* gene (*VHL*) is mutated or silenced by promoter methylation, leading to dysfunctional VHL protein and subsequent accumulation of the transcription factor Hypoxia Inducible Factor 1 (HIF1α) (4). This leads to a state of pseudohypoxia, characterized by expression of HIF-target genes that are responsible for stimulation of angiogenesis and cell survival (4–8). Based on this molecular insight angiogenesis inhibitors have been implemented as targeted agents for progressive ccRCC patients (9). One of the current choices for first-line treatment is sunitinib, a tyrosine kinase inhibitor (TKI) with activity against vascular endothelial growth factor receptors (VEGFRs) and platelet-derived growth factor receptor β (PDGFRβ), but also receptor tyrosine kinases PDGFRα, KIT, FLT3, RET, and CSF1R. Twenty percent of ccRCC patients with metastasized disease does not respond to this treatment, whereas another 30% develops resistance within 12 months (10). These patients are treated in second-line with other TKIs such as cabozantinib (targeting VEGFRs, MET, AXL, RET, KIT, FLT3), mTOR inhibitors, or immune checkpoint inhibitors (11–13). Rationale for treatment with these drugs comes from clinical trials, but therapy decision making in general does not include the molecular characteristics of an individual tumor. The high rate of non-responders and occurrence of serious side effects call for novel methods to determine optimal treatment protocols for individual m-ccRCC patients.

HIF1 hyperactivity also induces a shift in metabolism (14–16). Instead of shuffling glucose-derived pyruvate to the mitochondrial tricarboxylic acid (TCA) cycle, ccRCC cells convert pyruvate into lactate to accommodate their energy demand. The increased glucose uptake that accompanies this glycolysis also leads to increased activity of the pentose phosphate pathway (PPP), an important producer of nucleotides and reductive power. These metabolic alterations are strongly associated with disease progression and patient survival (14, 17). Altered metabolism may therefore be an appropriate therapeutic target for m-ccRCC, but carries a risk of side-effects in healthy tissues (18). We hypothesized that concomitant inhibition of tumor cell tyrosine kinases, angiogenesis and metabolism may have additive or even synergistic effects, and may allow dose reduction to minimize effects on healthy tissues.

Here, we determined expression levels of potentially actionable genes in clinical ccRCCs and in RCC cell lines using targeted RNA next generation sequencing (t/RNA-NGS) (16, 19), and show that from the transcriptional profiles potentially effective treatment combinations can be inferred.

## Materials and Methods

### Patient Material

Use of patient tissues for this study was approved by the local committee of the Radboudumc and involved informed consent. All methods were performed in accordance with the guidelines for use of human tissue of the Radboudumc. Immediately after tumor nephrectomy, one cm^3^ of healthy-appearing renal tissue (referred to as healthy tissue) and three tumor tissue fragments (T1-3) from different parts of the tumor were collected and snap-frozen in liquid nitrogen. Tissue samples were anonymized to the researchers. All tumors were identified as ccRCC by standard histopathology. Of all tissue samples H&E stainings were performed on 4 μm cryosections to estimate tumor cell percentage.

### Cell Culture

*VHL*-defective cell lines SKRC7 and SKRC17 are derived from a primary human ccRCC and a soft tissue metastasis of ccRCC, respectively (20). Both cell lines were cultured in RPMI 1640 medium (Lonza Group, Switzerland) supplemented with 10% fetal calf serum (FCS) (Gibco, Thermo Fisher Scientific, Waltham, MA, USA) and 40 μg/ml gentamycin (Centrafarm, Etten-Leur, The Netherlands) at 37°C in a 5% CO_2_ environment. The patient-derived astrocytoma cell line E98 has been described before (21) and was cultured in Dulbecco's Modified Eagle's Medium (DMEM) containing 4.5 g/L glucose and 4 mM L-glutamin (Lonza, Basel, Switserland), supplemented with 10% FCS (Gibco, Waltham, MA, USA) and 40 μg/ml gentamycin (Centrafarm, Ettenleur, The Netherlands) at 37°C in the presence of 5% CO_2_.

### Targeted RNA Sequencing

RNA was isolated from 10 μm cryosections using TRIzol reagent (ThermoFisher Scientific, Waltham, MA, USA) and reverse transcribed with Superscript II (ThermoFisher Scientific) using random hexamer primers, according to the manufacturer's instructions. Targeted RNA sequencing using smMIPs has been described before (16, 22). In short, smMIPs were designed against target regions of interest (UCSC human genome assembly hg19 and splice-variant specific FASTA sequences) based on the MIPgen algorithm as described by Boyle et al. (23), including a random octanucleotide unique molecule identifier (UMI). SmMIPs were phosphorylated using T4 polynucleotide kinase as described (16). The panel of transcripts of interest as presented in de Bitter et al. (16) was expanded with new cancer-related transcripts (Supplementary Table S1). Phosphorylated smMIPs (898 smMIPs, together targeting 150 transcripts) were hybridized to 50 ng of cDNA, followed by enzymatic gap-fill by primer extension and ligation. Exonuclease treatment, PCR amplification and library pooling and purification were performed as described (16). smMIP-PCR libraries were sequenced on the Illumina Nextseq platform (Illumina, San Diego, CA) at the Radboudumc sequencing facility. Reads were mapped against reference transcripts (UCSC human genome assembly hg19 and variant-specific FASTA sequences) using the SeqNext module of JSI SequencePilot version 4.2.2 build 502 (JSI Medical Systems, Ettenheim, Germany). The UMI was used to reduce all identical PCR amplification products to one consensus read originating from the same smMIP (unique read). Unique read counts for each smMIP were normalized to the total unique read count within a sample and multiplied by 10^6^ (Fragments per Million, FPM). Individual transcript levels were expressed as mean FPM of all smMIPs targeting that transcript. Variant calling was performed within SeqNext, excluding all variants with a coverage of <10% variant reads.

### Western Blot

Cryosections of normal kidney and matched tumor tissues were lysed in 1x RIPA buffer (Cell Signaling Technology, CST, Danvers, MA) supplemented with 1 mM phenylmethylsulfonyl fluoride (PMSF), according to manufacturer's instructions. Cell lines were seeded in 6-well plates and allowed to adhere. E98, SKRC7 and SKRC17 cells at ~80% confluence were treated with MET inhibitors Compound A [targeting MET; Amgen, Thousand Oaks, CA, USA (24, 25)], cabozantinib [targeting MET, VEGFR, RET, AXL, KIT, FLT3; Exelixis, San Francisco, CA, USA (26–28)] or DMSO vehicle for 20 min. Subsequently, SKRC7 cells were stimulated with HGF for 10 min. After treatment, cells were washed twice with ice-cold PBS and lysed in 1x RIPA buffer.

Cell lysates were subjected to electrophoresis on 10% SDS-PAGE gels and electroblotted onto nitrocellulose membranes (Whatman Optitran BA-S85, GE Healthcare, Little Chalfont, UK). Following blocking of aspecific binding sites in blocking buffer [1:1 PBS/Odyssey blocking buffer [LI-COR Biosciences, Lincoln, NE, USA]], blots were incubated o/n at 4°C with primary antibodies: rabbit-anti-MET (1:2,500, CST, #8198), rabbit-anti-P-MET Tyr1234/Tyr1235 (1:2,500, CST, #3077), rabbit-anti-P-ERK1/2 Thr202/Tyr204 (p44/p42, 1:500, CST, #4376), rabbit-anti-P-AKT S473 (1:2,500, CST, #4060), rabbit-anti-CAIX (1:1,000, Epitomics, #3829B), goat-anti-γ-tubulin C-20 (1:5,000, Santa crus, #sc-7396), and mouse-anti-GAPDH (1:10,000, Abcam, ab8245). Primary antibodies were detected with appropriate IRDye680- or IRDye800-conjugated secondary antibodies (Invitrogen Molecular Probes, Waltham, MA, USA) incubated 1 h at RT shielded from light. Signals were visualized using the Odyssey imaging system (LI-COR Biosciences, Lincoln, NE, USA).

### Cell Proliferation Assays

Cells were seeded in 96-well plates (2,000 and 20,000 cells/well for SKRC7/SKRC17 and E98 cells, respectively). The next day increasing concentrations of Compound A, cabozantinib, gefitinib (targeting EGFR; Axon Medchem, Groningen, The Netherlands) or 6-aminonicotinamide (6AN, targeting glucose-6-phosphate dehydrogenase (G6PD); Sigma-Aldrich, St. Louis, MO, USA) were added to the medium. For monotherapy assays with Compound A and cabozantinib metabolic activity of cells was measured 4 days later by incubation with 0.5 mg/ml 3-(4,5-dimethylthiazol-2-yl)-2,5-diphenyltetrazolium bromide (MTT) in PBS (Sigma-Aldrich, St. Louis, MO, USA). After 3.5 h incubation at 37°C formazan crystals were dissolved in DMSO and optical densities were measured at 560 nm. Alternatively, for combination therapies total protein content was measured 4 days after start of treatment. Cells were washed with PBS and fixed overnight with 10% (w/v) trichloroacetic acid at 4°C. Total cellular protein was stained with 0.5% (w/v) sulfurhodamine B (SRB, Sigma-Aldrich, St. Louis, MO, USA) in 1% acetic acid. After 20 min wells were washed 4 times with 1% acetic acid to remove unbound dye and dried at 60°C. Protein-bound SRB was solubilised using 150 μl 1 mM Tris-HCl (pH = 10) and optical densities were measured at 560 nm.

Synergy of drug combinations was assessed by calculation of the combination index (CI) and dose reduction index (DRI) using CompuSyn software (ComboSyn, Inc.), according to the manufacturer's instructions (29, 30). Increasing concentrations of Compound A and cabozantinib were combined with gefitinib and 6AN in a constant ratio. Levels of synergy were calculated using the fraction affected (FA-) value, and classified as follows: CI = 0.1–0.3 strong synergism, CI = 0.3–0.7 synergism, CI = 0.7–0.85 moderate synergism, CI = 0.85–0.9 slight synergism, CI = 0.9–1.1 additive, CI = 1.1–1.2 slight antagonism, CI = 1.2–1.45 moderate antagonism, CI = 1.45–3.3 antagonism. The DRI denotes an indication for the fold of dose-reduction allowed for each drug due to synergism when compared with the dose of each drug alone.

### Statistical Analysis

Statistical analyses were performed in R (version 3.4.3). Mean FPM values were log_2_ transformed (after addition of 0.01 to prevent log(0) transformation errors) and clustered in an unsupervised manner using the Manhattan distance and Average (Unweighted Pair Group Method with Arithmetic Mean, UPGMA) clustering method, and translated into a heatmap. A Wilcoxon Mann-Whitney *U* test was performed to find differentially expressed genes between clusters (*p* < 0.05). Multiple testing corrections were done using Benjamini Hochberg (FDR < 0.01).

## Results

**Figure 1:**
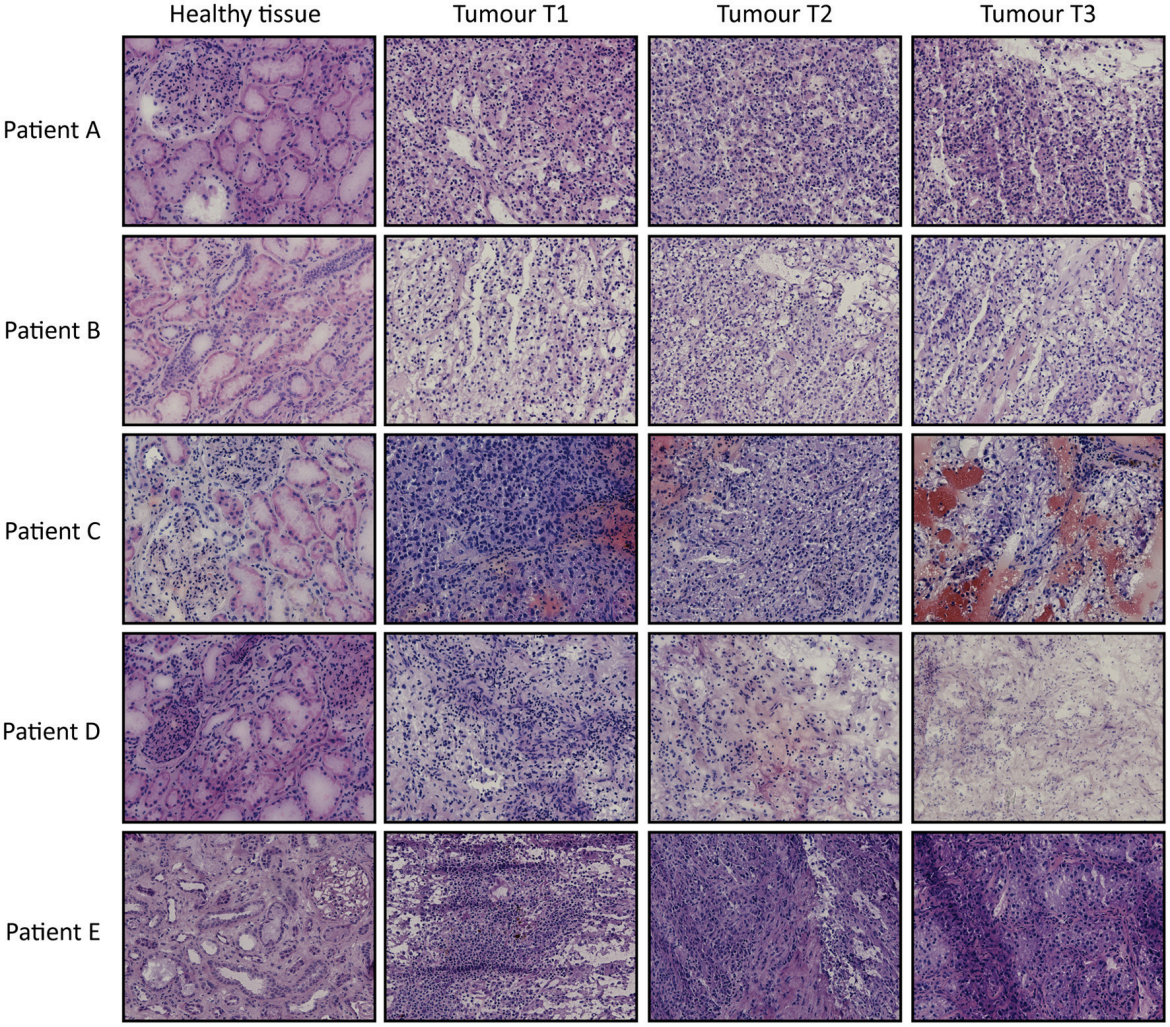
H&E stainings of tissues biopsies from ccRCC patients A–E. For each patient one healthy-appearing kidney sample and three tumor biopsies (T1–T3) are included. Original magnification 20×.

**Figure 2:**
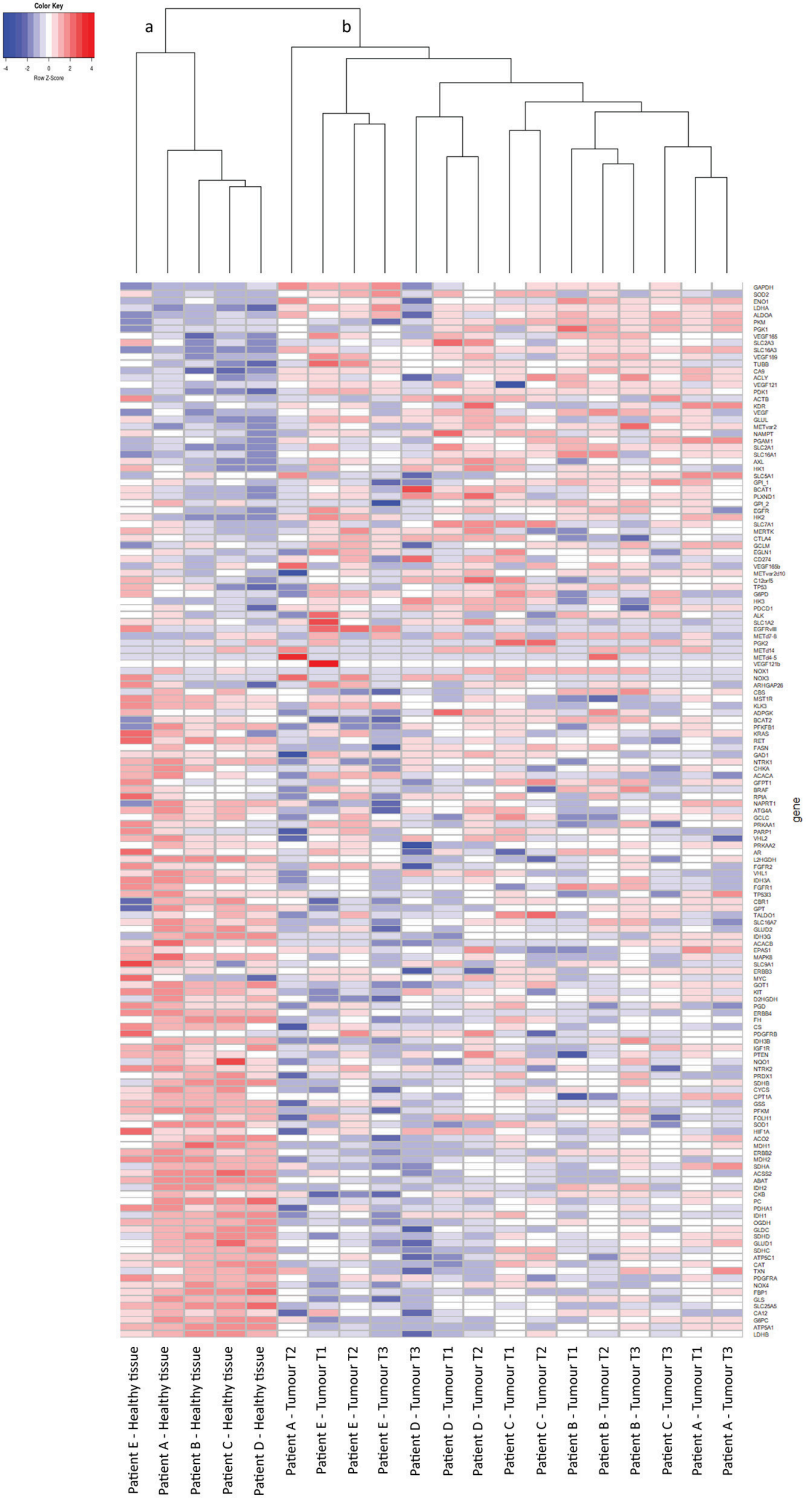
t/RNA-NGS of ccRCC and healthy kidney tissue. Tissues originate from five ccRCC patients, one healthy-appearing kidney sample and three tumor biopsies each. Heatmap containing 152 genes, generated by unsupervised hierarchical clustering using the Manhattan distance and Average clustering method. Two head clusters are generated: cluster **(a)** contains all healthy kidney tissues, while cluster **(b)** consists of the tumor biopsies from all five ccRCC patients. The three tumor biopsies from patients B, D, and E cluster together in a subcluster, while patients A and C both have one tumor sample that groups separately from the other two.

From 5 tumor nephrectomies, one biopsy of healthy kidney tissue and three matched tumor biopsies were collected for t/RNA-NGS. H&E staining of all tumor samples confirmed ccRCC diagnosis. Normal-appearing kidney tissues, taken at distance of the tumor, were free of cancer cells (Figure 1). The mean unique read count of t/RNA-NGS per sample was >10^6^, which is sufficient to generate reliable expression and mutation data. Unsupervised hierarchical clustering of gene expression levels (given as Fragments per Million, FPM) of all 20 samples as obtained with t/RNA-NGS resulted in two main clusters a and b, comprising all healthy kidney tissues and all tumor tissues, respectively (Figure 2). Raw data as FPM for all tissue samples are shown in Supplementary Table S3 and for cell lines in Supplementary Table S4. Tumor biopsies from patients B, D, and E clustered together in subgroups, showing that intertumor variability for these patients was higher than intratumor heterogeneity. For patients A and C, one of the three tumor samples grouped separately from the other two.

**Figure 3:**
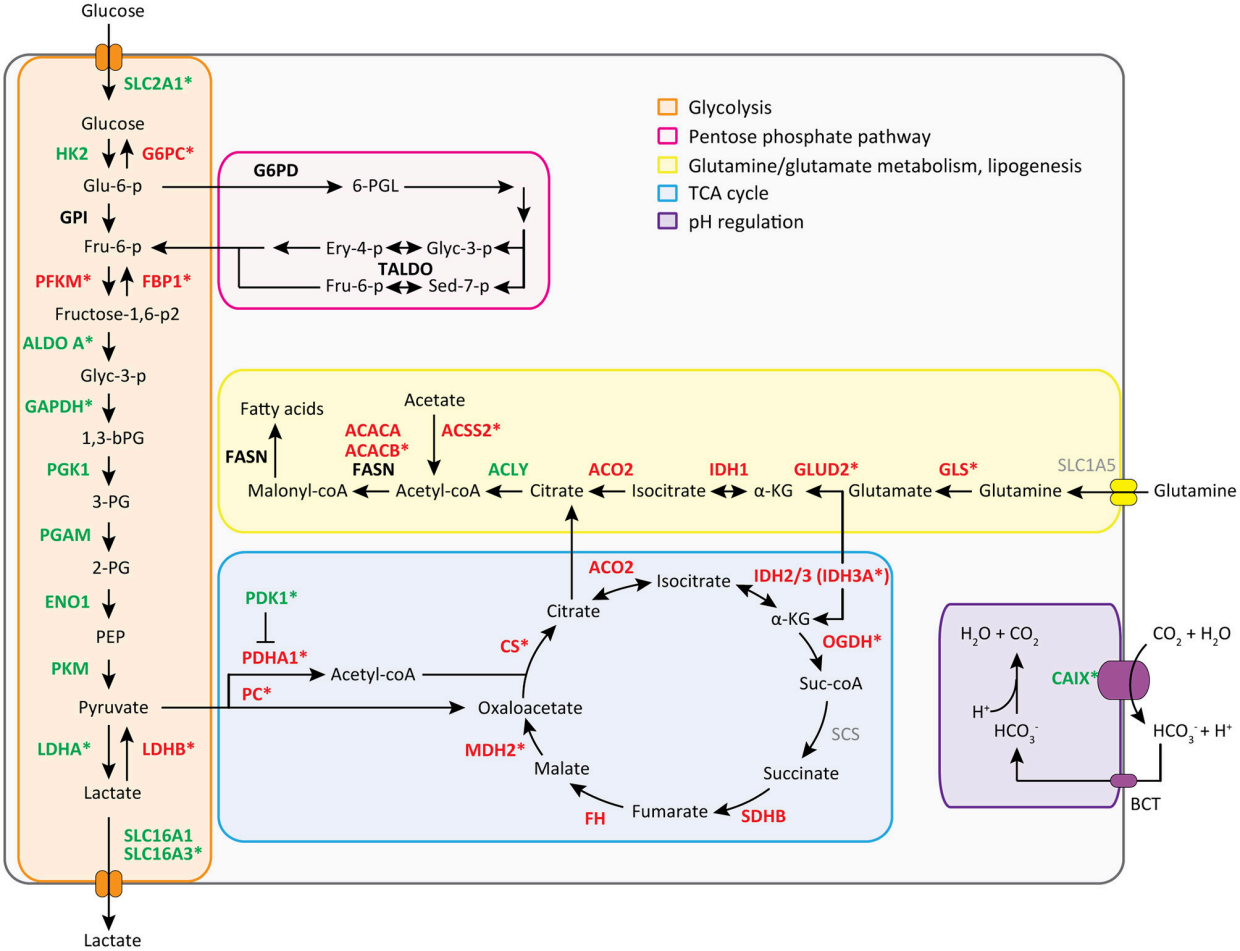
Metabolic transcript expression changes in ccRCC tissue compared to healthy kidney tissue. Mean FPM values of cluster a (healthy-appearing kidney tissues) were compared with mean FPM values of cluster b (tumor biopsies) using the fold change of (cluster b/a) or −1/(cluster b/a). For visuality, transcripts with a fold change of >1.5 or <-1.5 (see Table 1 and Supplementary Table S2) are colored in green and red, respectively. Transcripts marked with a ^*^ are changed significantly, as determined with a Wilcoxon Mann-Whitney *U* test and Benjamini Hochberg correction for multiple testing (*p* < 0.05, FDR < 0.05) (see Table 1 and Supplementary Table S2).

**Figure 4:**
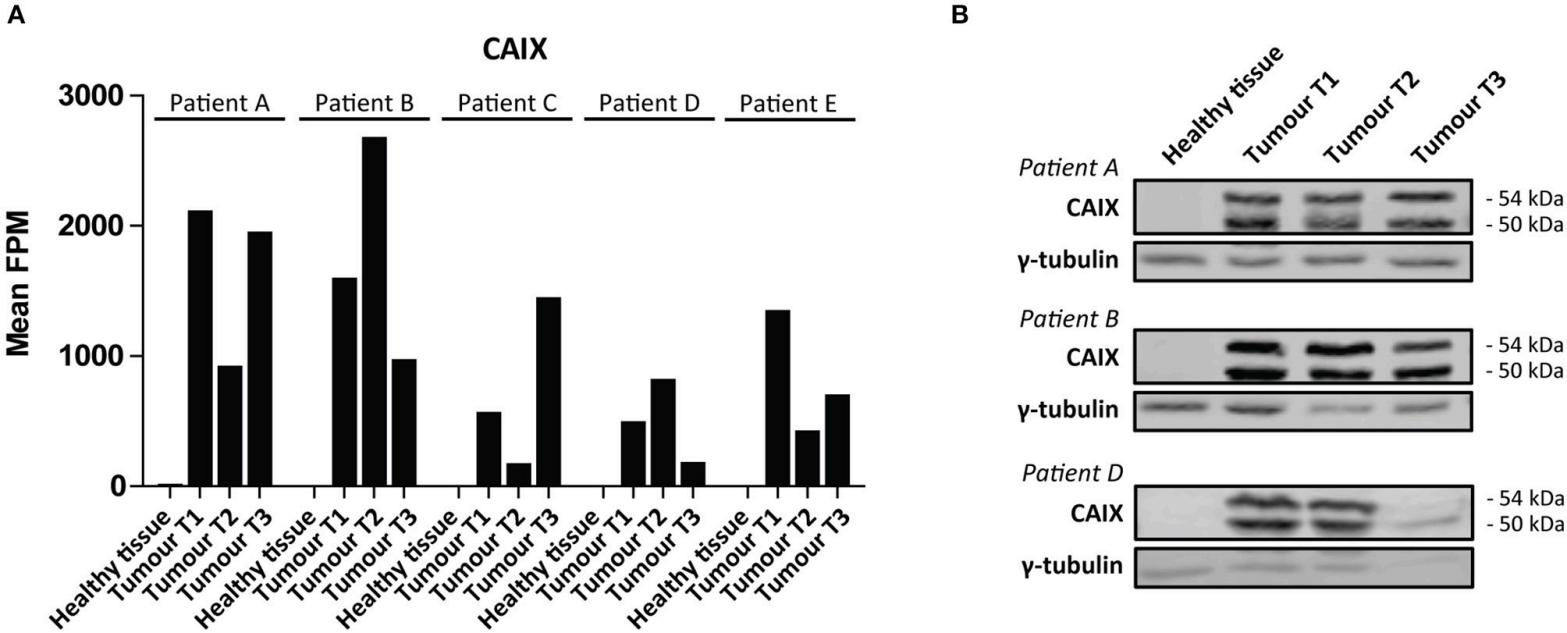
CAIX expression in ccRCC. **(A)** CAIX FPM values in each individual tissue biopsy of ccRCC patients A-E. CAIX expression is absent in healthy kidney tissues, while elevated in ccRCC tissues. **(B)** CAIX transcript expression levels correlate well with protein levels, as shown for three representative patients. γ-tubulin was used as a loading control. Presented blots are cropped.

We then performed a Wilcoxon Mann-Whitney *U* test to compare gene expression profiles in cluster a vs. cluster b. Expression of 44 transcripts differed significantly between both clusters (Table 1). Expression levels and fold changes of all genes of which expression did not significantly differ are outlined in Supplementary Table S2. Differential gene expression was observed for genes associated with glucose import and glycolysis (high in cluster b, ccRCC tissues), and genes encoding TCA cycle enzymes, glutamine/glutamate metabolism and lipid synthesis (low in cluster b) (Figure 3). HIF1 target genes carbonic anhydrase IX (CAIX) and the pro-angiogenic VEGF-A isoform VEGF165 were significantly associated with cluster b. CAIX levels of all tissue biopsies are presented in Figure 4A. CAIX transcript expression correlated well with protein levels (representative examples shown in Figure 4B). Transcript levels of tyrosine kinase receptors ERBB4, PDGFRA, ERBB2, RET, KIT, NTRK1, MST1R (RON), and NTRK2 were significantly decreased, while expression of the tyrosine kinase receptor MET was significantly increased in cluster b compared to cluster a (Table 1).

**Table 1:** Differential gene expression in cluster a vs. cluster b.

**Gene**	**Mean FPM cluster a**	**Mean FPM cluster b**	***P*-value**	**FDR**	**FC cluster b/a**	**Significant?**
ABAT	712.42	32.62	0.000	0.000	−21.84	Y
ATP5A1	6464.66	2144.48	0.000	0.000	−3.01	Y
CAIX	5.13	1097.27	0.000	0.000	214.06	Y
ERBB4	252.77	3.68	0.000	0.000	−68.61	Y
GLDC	1238.58	139.98	0.000	0.000	−8.85	Y
GSS	792.49	370.62	0.000	0.000	−2.14	Y
L2HGDH	111.61	24.42	0.000	0.000	−4.57	Y
LDHB	7589.19	2865.01	0.000	0.001	−2.65	Y
MAPK8	311.14	141.16	0.000	0.001	−2.20	Y
MDH2	1060.56	496.07	0.000	0.001	−2.14	Y
NOX4	2607.55	533.81	0.000	0.001	−4.88	Y
OGDH	1526.43	594.58	0.000	0.001	−2.57	Y
PDGFRA	2089.59	222.00	0.000	0.001	−9.41	Y
PDHA1	1245.83	410.41	0.000	0.001	−3.04	Y
PDK1	130.28	787.14	0.000	0.001	6.04	Y
SLC16A3	98.64	1710.95	0.000	0.001	17.34	Y
SLC25A5	4646.10	1162.87	0.000	0.001	−4.00	Y
SLC2A1	56.63	356.51	0.000	0.001	6.30	Y
ACACB	274.19	113.33	0.000	0.001	−2.42	Y
ACSS2	873.58	240.91	0.000	0.001	−3.63	Y
ERBB2	789.49	225.30	0.000	0.001	−3.50	Y
FBP1	2948.42	301.68	0.000	0.001	−9.77	Y
GLS	6088.25	3153.33	0.000	0.002	−1.93	Y
GOT1	295.00	104.45	0.000	0.002	−2.82	Y
IDH3A	223.68	126.11	0.000	0.002	−1.77	Y
LDHA	2011.93	9899.14	0.000	0.002	4.92	Y
PFKM	653.08	223.63	0.000	0.002	−2.92	Y
RET	12.48	1.54	0.000	0.002	−8.08	Y
CAXII	7537.68	3708.13	0.001	0.002	−2.03	Y
CAT	2411.16	923.26	0.001	0.002	−2.61	Y
CS	679.43	421.53	0.001	0.002	−1.61	Y
G6PC	4206.01	110.88	0.001	0.002	−37.93	Y
KIT	302.27	99.36	0.001	0.002	−3.04	Y
PC	914.08	95.52	0.001	0.002	−9.57	Y
NTRK1	22.30	1.50	0.001	0.002	−14.88	Y
ATP5C1	3487.96	2282.76	0.002	0.002	−1.53	Y
MST1R	5.38	1.86	0.002	0.002	−2.89	Y
NTRK2	455.23	104.78	0.002	0.003	−4.34	Y
SLC16A1	225.93	506.49	0.002	0.003	2.24	Y
ALDOA	6340.23	13415.21	0.002	0.003	2.12	Y
GAPDH	15645.76	26590.07	0.002	0.003	1.70	Y
GLUD2	238.29	95.90	0.002	0.003	−2.48	Y
MET	351.99	727.90	0.002	0.003	2.07	Y
VEGF165	411.68	2078.47	0.002	0.003	5.05	Y

*Expression of 44 genes differed significantly between cluster a and cluster b. Fold changes of cluster b/cluster a are indicated. Significance was determined using a Wilcoxon Mann-Whitney U test with Benjamini Hochberg correction for multiple testing (p < 0.05, FDR < 0.01). Note that for significance, the p-value must not exceed the FDR. Fold changes are calculated as (cluster b/a), or -1/(cluster b/a) to prevent fold changes >0 and <1. FC, fold change*.

Variant calling of the t/RNA-NGS datasets revealed mutations in the VHL gene in tumor samples of four of the patients, which were not present in matched healthy kidney tissue, identifying these as somatic mutations. Three patients had VHL STOP mutations whereas in patient C the known pathogenic VHL p.L118P mutation was detected in one tissue biopsy, in 29% of the reads (Table 2). The coverage of the corresponding c.353T locus in this sample was 22 unique reads as compared with 7 and 18 unique read counts in the other two biopsies of the same tumor, in which the mutation was not called. This suggests intratumor heterogeneity of this pathogenic variant, although low coverage may also cause a false-negative result.

**Table 2:** VHL mutations in ccRCC.

**Patient**	**Biopsy**	**Variant reads (%)**	**AA change**	**c. HGVS**	**p. HGVS**
A	Healthy tissue	–			
	Tumor T1	–			
	Tumor T2	25%	[STOP] AA 130 (E2/48)	c.246_247delCG	p.Val83Argfs^*^48
	Tumor T3	16%	[STOP] AA 130 (E2/48)	c.246_247delCG	p.Val83Argfs^*^48
B	Healthy tissue	–			
	Tumor T1	–			
	Tumor T2	58%	[STOP] AA 158 (E3/9)	c.369delG	p.Thr124Hisfs^*^35
	Tumor T3	–			
C	Healthy tissue	–			
	Tumor T1	29%	L -> P (118)	c.353T>C	p.Leu118Pro
	Tumor T2	–			
	Tumor T3	–			
D	Healthy tissue	–			
	Tumor T1	–			
	Tumor T2	–			
	Tumor T3	–			
E	Healthy tissue	–			
	Tumor T1	57%	[STOP] AA 173 (E3/54)	c.457dupC	p.Leu153Profs^*^21
	Tumor T2	28%	[STOP] AA 173 (E3/54)	c.457dupC	p.Leu153Profs^*^21
	Tumor T3	26%	[STOP] AA 173 (E3/54)	c.457dupC	p.Leu153Profs^*^21

*Different VHL mutations with variable coverage levels were detected in four of the five ccRCC patients, with VHL STOP mutations in three of the patients*.

**Figure 5:**
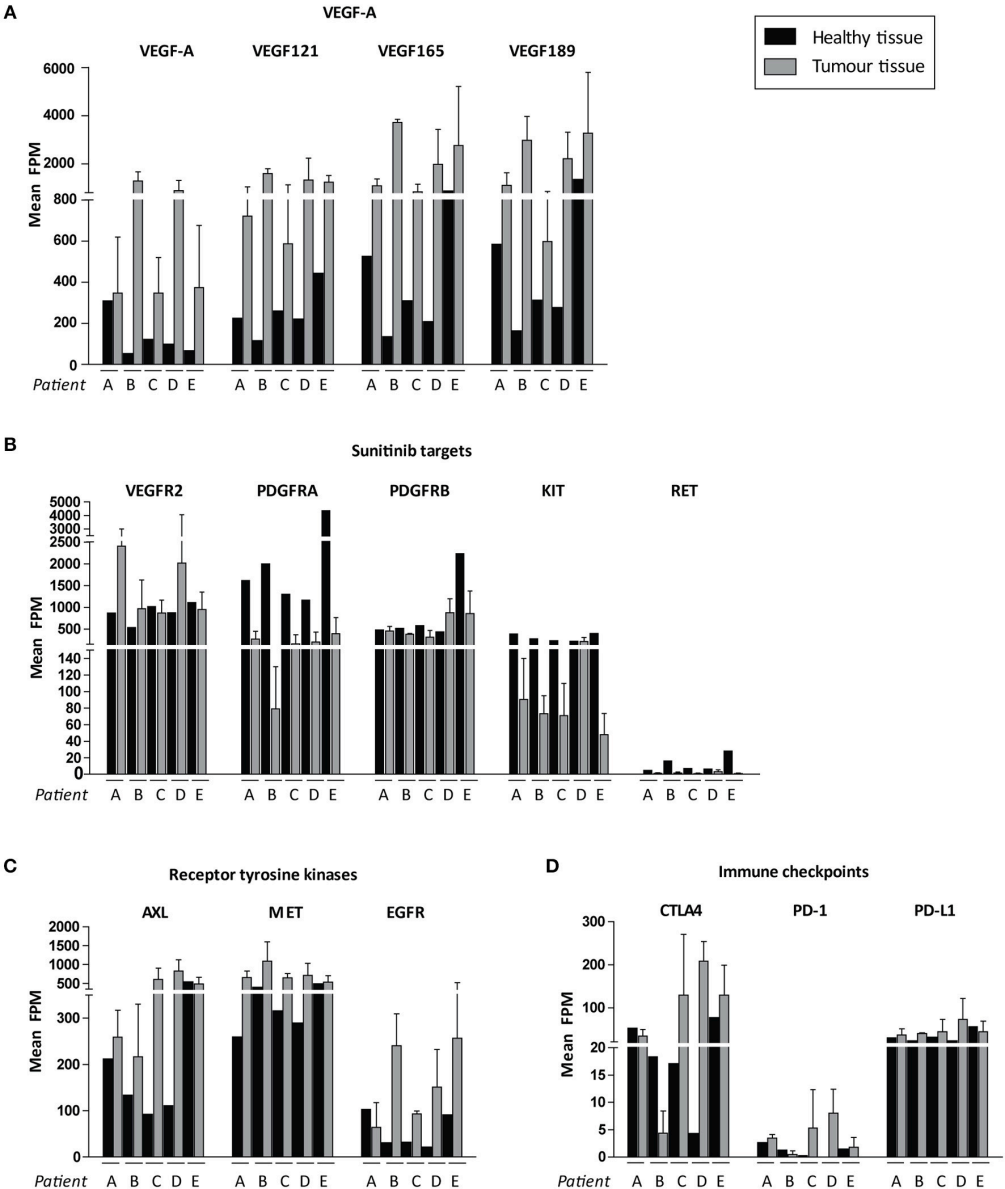
Possible targets for individualized targeted therapeutics. Expression levels are shown in FPM. FPM levels of the three tumor biopsies for each patient are averaged. Expression of **(A)** VEGF-A variants, **(B)** sunitinib target genes, **(C)** other targetable receptor tyrosine kinases, **(D)** immune checkpoints in ccRCC patients A–E. Inter-tumor heterogeneity is visualized. Data represent mean ± SD.

We then focussed on the relative expression levels of tyrosine kinases that are potential targets for available precision medicines. VEGF-A and the pro-angiogenic isoforms VEGF121, VEGF165, and VEGF189 were highly expressed in all cancers, although there was interpatient heterogeneity with respect to VEGFR2 expression levels (Figures 5A,B). Of note, PDGFRA and KIT were expressed at relatively low levels in tumor tissue and higher in healthy kidney tissue (Figure 5). Among other targetable tyrosine kinases AXL, MET and EGFR were expressed at high levels in some, but not all, tumor samples (Figure 5C). Expression of cytotoxic T-cell marker CTLA4 varied widely between patients, whereas expression of PD-1 (PDCD1) was uniformly low and tumor cell marker PD-L1 (CD274) was only moderately expressed (Figure 5D).

**Figure 6:**
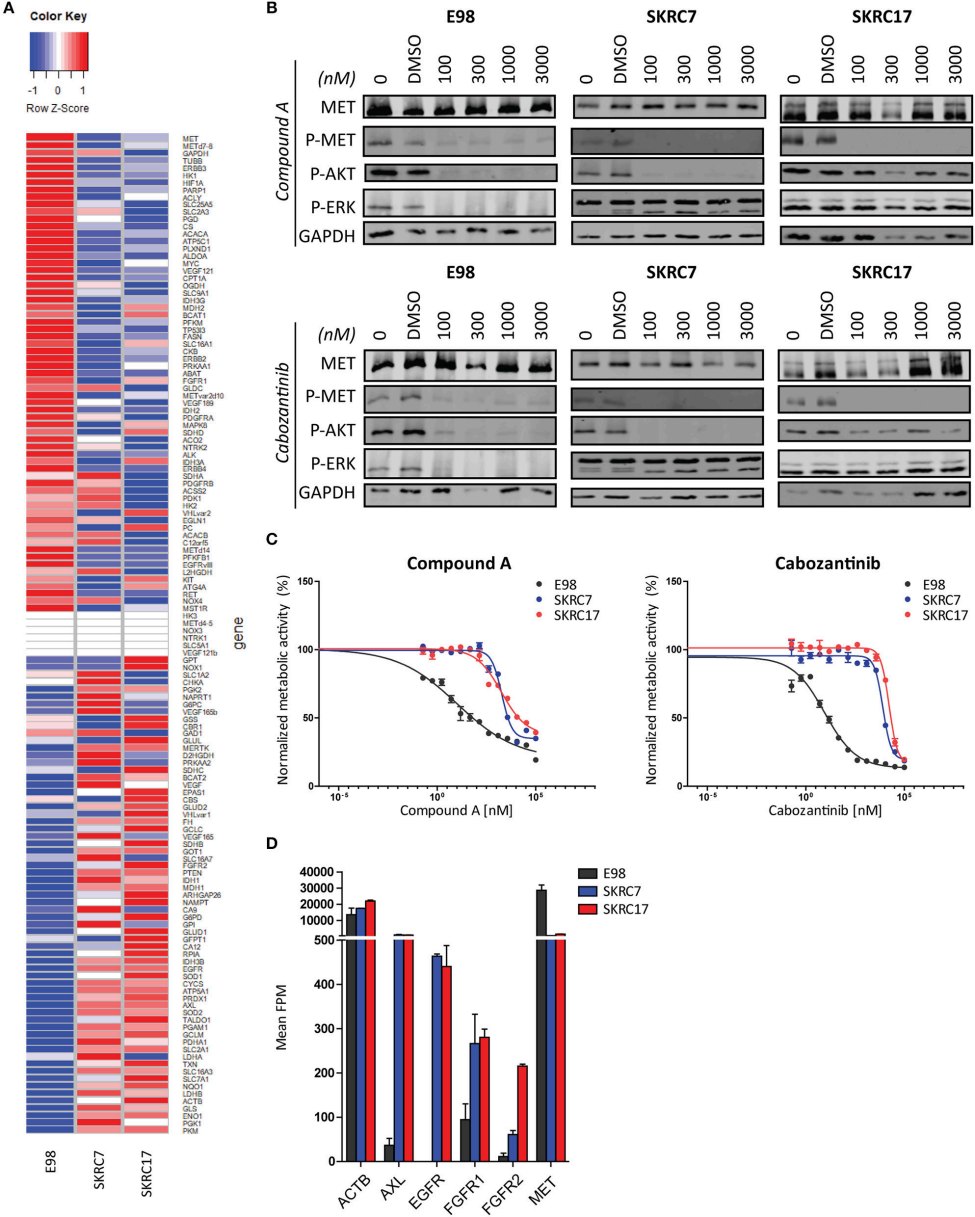
Targeted therapy prediction based on t/RNA-NGS. **(A)** Heatmap showing expression levels (FPM) of 136 genes in E98, SKRC7 and SKRC17 cell lines. **(B)** E98, SKRC7 and SKRC17 cells were treated for 20 min with Compound A or cabozantinib. SKRC7 cells were stimulated with HGF for 10 min subsequently. Phosphorylation levels of MET (Tyr1234/Tyr1235), AKT (Ser473), and ERK1/2 (Thr202/Tyr204) were monitored by western blot, with GAPDH as a loading control. Presented blots are cropped. **(C)** MTT proliferation assays of E98, SKRC7 and SKRC17 treated with Compound A or cabozantinib. Both compounds show high efficacy in E98, but less in SKRC7 and SKRC17 cells. Data represent mean ± SD (*N* = 4). **(D)** FPM expression levels of a selection of tyrosine kinases in E98, SKRC7, and SKRC17. While E98 shows high expression of only the tyrosine kinase MET, SKRC cell lines also have considerable expression of AXL, EGFR, FGFR1, and FGFR2, tyrosine kinases that may be responsible for resistance to MET inhibition in these cells. Data represent mean ± SD (*N* = 2).

To test the hypothesis that t/RNA-NGS datasets can be used to guide targeted therapy we performed smMIP-based targeted RNA sequencing on patient-derived renal carcinoma cell lines SKRC7 and SKRC17 (Figure 6A). These cell lines express MET transcript and protein at levels comparable to the E98 astrocytoma cell line (21) (Figures 6A,B), which is very well-characterized by our group (21, 25, 28) and was used as a positive control. MET phosphorylation in SKRC17 cells did not require addition of exogenous HGF, possibly a result of endogenous production of HGF (as determined by w/RNA-NGS, not shown). E98 cells were sensitive to MET inhibition by the multi-kinase inhibitor cabozantinib and mono-inhibitor Compound A as shown by decreased levels of phosphorylated MET (25, 28). The same effects on pMET were observed in SKRC7 and SKRC17 cells. However, whereas in E98 cells decreased inhibition of MET phosphorylation coincided with decreased levels of pAKT and pERK, effects on pAKT were only minor in SKRC17 and pERK levels were unchanged in both cell lines (Figure 6B). In E98 cells inhibition of MET by Compound A or cabozantinib translated to decreased proliferation rates, but this was not the situation in SKRC7 and SKRC17 cells (Figure 6C). A possible explanation is expression of additional membrane tyrosine kinases AXL, EGFR, and FGFR1/2 that signal via similar pathways as MET. These kinases are not expressed by E98 and may provide compensation pathways for MET inhibition (Figures 6A,D).

**Figure 7:**
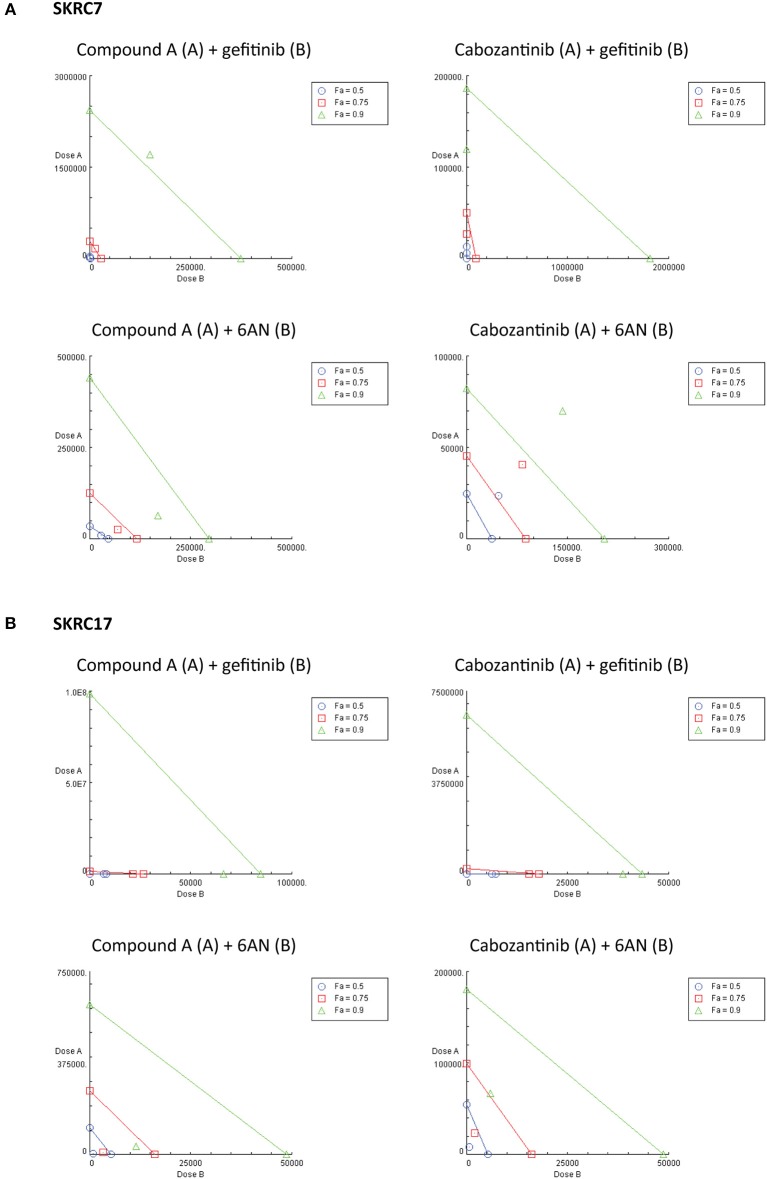
Combination therapy of MET inhibitors Compound A and cabozantinib with an EGFR inhibitor (gefitinib) or G6PD inhibitor (6AN) in **(A)** SKRC7 and **(B)** SKRC17 cell lines. Drug effects were tested using SRB assays. The FA-value represents the fraction of cell viability affected by therapy. Points on the graph axes represent the dose of the monotherapy (drug A or B, as indicated in the figure head of each isobologram) necessary to generate the given FA-value. The line connecting the x- and y-axis represents an additive effect (CI = 1) of the combination at the given FA-value. Points below or above the line represent synergism (CI < 1.0) or antagonism (CI > 1.0), respectively (*N* = 2).

Considering the expression of potential rescue kinases in SKRC7 and SKRC17 cells and the known interplay of MET and EGFR in therapy resistance (25), we tested combinations of the MET inhibitors Compound A and cabozantinib with the EGFR inhibitor gefitinib on SKRC7 and SKRC17 cell viability. Gefitinib induced moderate levels of synergy when combined with the MET inhibitor Compound A in SKRC17, but not SKRC7 cells. The combination of the MET/AXL/VEGFR2 inhibitor cabozantinib with gefitinib acted synergistically in SKRC7, but less so in SKRC17 cells (Figure 7 and Table 3).

**Table 3:** Combination index (CI) and dose reduction index (DRI) values for the combination therapies as indicated, in SKRC7 and SKRC17 cell lines.

	**SKRC7 CI (DRI drug A;DRI drug B)**			**SKRC17 CI (DRI drug A;DRI drug B)**		
	**FA 0.5**	**FA 0.75**	**FA 0.9**	**FA 0.5**	**FA 0.75**	**FA 0.9**
Compound A (drug A) + gefitinib (drug B)	1.07 (2.2;1.6)	1.06 (1.8;2.0)	1.1 (1.4;2.5)	0.85 (35.8;1.2)	0.8 (642.6;1.3)	0.78 (1528.2;1.3)
Cabozantinib (drug A) + gefitinib (drug B)	0.49 (2.2;41.9)	0.55 (1.8;177.0)	0.64 (1.6;748.7)	0.94 (11.01;1.2)	0.88 (120.0;1.2)	0.89 (1307.0;1.1)
Compound A (drug A) + 6AN (drug B)	0.93 (3.3;1.6)	0.81 (4.8;1.7)	0.72 (6.9;1.7)	0.21 (39.5;5.5)	0.24 (27.0;4.8)	0.29 (18. 5;4.2)
Cabozantinib (drug A) + 6AN (drug B)	2.21 (1.1;0.8)	1.84 (1.1;1.1)	1.55 (1.2;1.4)	0.29 (6.7;7.3)	0.37 (4.2;7.7)	0.49 (2.7;8.2)

*The FA-values represent the fraction of cell viability affected by treatment. CI and DRI values are given for FA values of 0.5, 0.75, and 0.9*.

Because activity of TKIs does not only depend on expression levels of tyrosine kinases, but also on the expression levels and activities of the counteracting phosphatases (31), reliable prediction of therapeutic efficacy of drug combinations is highly complex. Given the characteristic metabolic alterations induced in ccRCC, we next investigated the effect of combination therapy of Compound A or cabozantinib with metabolic inhibition by 6AN, an inhibitor of the rate-limiting PPP intermediate G6PD (FPM 945.06 and 1241.78 in SKRC7 and SKRC17, respectively). In SKRC7 cells, combination of Compound A with 6AN induced moderate levels of synergism. Surprisingly, the combination of 6AN with cabozantinib was antagonistic in SKRC7 cells. However, combination of 6AN with Compound A or cabozantinib induced strong synergy in SKRC17 cells (Figure 7 and Table 3). All dose reduction indexes (DRI) for synergistic or additive drug combinations were favorable (Table 3).

## Discussion

Patients with m-ccRCC are treated by first-line anti-angiogenesis therapy upon signs of tumor progression and immune checkpoint or alternative TKIs in second-line (9). However, these therapies do not take into account the molecular characteristics of the individual tumor. Using t/RNA-NGS we here show that whereas the five ccRCCs in our study share the metabolic reprogramming characteristic for this cancer, expression of targetable tyrosine kinases is heterogeneous between tumors. Remarkably, PDGFRα and KIT are expressed at much higher levels in the normal kidney than in the tumor cells, showing that targeting of ccRCC by sunitinib is not as cancer-specific as desirable, which may explain observed sunitinib-induced toxicity. Because t/RNA-NGS allows a comprehensive overview of all druggable tyrosine kinases in a tumor, it may allow more rational therapy decision making than is currently possible. Interestingly, we found significantly elevated tyrosine kinase receptor MET levels in ccRCC tissues compared to healthy kidney tissue, in agreement with *in vitro* studies and ccRCC tissue analyses demonstrating upregulation of MET induced by inactivation of VHL (32, 33). There are multiple options for first-line TKI treatment including sunitinib, pazopanib, and sorafenib, all having a different target spectrum. Recently, also cabozantinib has been approved for first-line therapy of advanced RCC. Considering the high MET expression in our subset of untreated ccRCC patients, MET inhibitors cabozantinib (26–28) or Compound A (24, 25) may be well-suited for first-line treatment in this patient group. Furthermore, MET upregulation has been associated with the development of resistance to VEGFR inhibition (34). Multi-kinase inhibition of MET, AXL, and VEGFR2 by cabozantinib may therefore particularly be effective in ccRCCs with elevated expression levels of MET and AXL, to simultaneously interfere with development of therapy resistance. On the other hand, angiogenic ccRCCs that overexpress EGFR may benefit better from the EGFR/VEGFR2 inhibitor vandetanib. It is important to realize that transcriptome data do not always correlate with protein expression, and therefore proof of concept for transcription-based therapy prediction has to come from retrospective studies in which t/RNA-NGS profiles are analyzed with computational biology methods in relation to treatment and clinical outcome.

Dysfunctional VHL due to mutations or promoter hypermethylation are known drivers of ccRCC, causing accumulation of HIF1 and its target genes (4–8). In all five patients we confirmed VHL-associated metabolic alterations, notably upregulation of expression of genes involved in glycolysis and a decrease of expression levels of genes involved in the TCA cycle (14, 15, 35). In line with previous studies (36–38) expression of FBP1 was significantly decreased. FBP1 loss has been reported as a second unique feature of RCC and a mediator of HIF1-induced metabolic changes (39, 40). Nonetheless, VHL mutations were detected in only four of the five ccRCC patients, and not in each of three biopsies. Low unique read coverage of transcript locations corresponding to a mutation may cause false-negative variant calling. Moreover, it has recently been suggested that a range of four to eight biopsies is required to capture the majority of driver events (>75%) in ccRCC (41), therefore sampling may also explain variability in mutation detection. Another possible explanation for failure to detect aberrant VHL is promoter hypermethylation, which was outside the scope of this study.

While other studies have demonstrated increased expression of genes involved in PPP, glutamine metabolism and fatty acid synthesis (14, 17), we found a significantly decreased expression of most enzymes involved in glutamine/glutamate metabolism and fatty acid synthesis in ccRCC tissues compared to healthy kidney tissue. Whereas dual activation of HIF2 and MYC induces glutamine-dependent lipogenesis in RCC (14, 42–44), levels of both HIF2 and MYC expression in these five ccRCC patients were unaltered, possibly explaining the absence of increased glutamine synthesis. Moreover, the presence of metabolic shifts depends on aggressiveness of the tumor. Low expression levels of GPI and G6PD, which were unaltered in tumor compared with healthy tissue, have been associated with better patient survival (45). The low disease stage of these five ccRCC patients possibly implies that they do not have high metastatic capacity, which may explain why enzymes in abovementioned pathways are not elevated.

Our *in vitro* data show that expression of therapy targets cannot always be translated to therapy response. Our data suggests that analysis involving the presence of direct therapy targets combined with the expression of genes in possible resistance pathways may allow prediction of therapy response. For example, MET and EGFR are well-known to cause mutual cross-resistance to targeted therapy in different cancer types (46–52). Combined inhibition of MET and EGFR *in vitro* in SKRC7 and SKRC17 cell lines showed additive and moderately synergistic effects, but with positive DRI values, suggesting that in combination therapy the doses of each drug may be decreased to reduce drug toxicity. The very high DRI values of Compound A and cabozantinib in SKRC17 indicate that the dosages of these drugs could be dramatically decreased to achieve the therapeutic effect of the combination treatment with gefitinib, although this should be confirmed by similar *in vitro* and additional *in vivo* studies. Excessive toxicity has shown to complicate drug combination therapies in patients, but some clinical trials have also reported promising results (53–56). Better prediction for selected application of targeted (combination) therapies is essential to design treatment strategies with maximal efficacy and minimal toxicity.

Transcript expression level-based combination therapy of MET tyrosine kinase inhibition with 6AN, a metabolic inhibitor of G6PD, did show strong synergism and positive DRI indexes in SKRC17 and may therefore be an interesting opportunity for new therapies. In SKRC7 the combination of 6AN with cabozantinib was however antagonistic, suggesting this combination of drugs may activate compensation pathways in these cells. Whereas, SKRC7 and SKRC17 are derived from a primary human ccRCC and a soft tissue metastasis of ccRCC, respectively, here combination treatment involving PPP targeting seems most interesting in m-ccRCC cells. These data mark the complexity of tumor biology and the need for cautiousness with targeted therapeutics.

Currently, ccRCC patients are offered first-line therapy based on clinical characteristics, and molecular characteristics are not included in the clinical management. Our data demonstrate the prominent inter-tumor variability that exists between ccRCC patients, and highlights the need for individual tumor profiling and personalized therapy. Moreover, t/RNA-NGS may allow repurposing of drugs that have been approved for other cancers but not (yet) for ccRCC.

## Data Availability

Datasets are available on request.

## Author Contributions

CH and WL designed the study. CH, CZ, and TB performed experiments. CH, AE, and MH analyzed t/RNANGS data. CH, PM, EO, and WL were involved in patient tissue collection. CH wrote the manuscript. All authors were involved in discussions and manuscript revision and approved the submitted version.

### Conflict of Interest Statement

The authors declare that the research was conducted in the absence of any commercial or financial relationships that could be construed as a potential conflict of interest.

## Supplementary Material

Supplementary Table 1 Transcripts for smMIP design. SmMIPs were designed against the antisense strand of predicted transcripts (UCSC human genome assembly hg19 and splice variant specific FASTA sequences). The genes listed are newly added to the panel published in de Bitter et al. (16).

Supplementary Table 2 Gene expression in cluster a vs. cluster b (non-significant). Fold changes of cluster b/cluster a are indicated. Significance was determined using a Wilcoxon Mann-Whitney U-test with Benjamini Hochberg correction for multiple testing (*p* < 0.05, FDR < 0.01). Note that for significance, the p-value must not exceed the FDR. Significant differential gene expression in cluster a vs. cluster b is shown in Table 1. Fold changes are calculated as (cluster b/a), or -1/(cluster b/a) to prevent fold changes >0 and <1. FC, fold change.

Supplementary Table 3 Mean FPM values for clinical tissue samples.

Supplementary Table 4 Mean FPM values for cell lines E98, SKRC7, and SKRC17.
