# Supplementary material for: Molecular Profiling of Druggable Targets in Clear Cell Renal Cell Carcinoma Through Targeted RNA Sequencing
Source: Front Oncol. 2019 Mar 1;9:117. doi: 10.3389/fonc.2019.00117 (PMC6407434; doi:10.3389/fonc.2019.00117)
Supplement: Supplementary Table 1 — Transcripts for smMIP design. SmMIPs were designed against the antisense strand of predicted transcripts (UCSC human genome assembly hg19 and splice variant specific FASTA sequences). The genes listed are newly added to the panel published in de Bitter et al. (16). [file Table_1.pdf]

Supplementary Table SI. Transcripts for smMIP design. SmMIPs were designed against the antisense strand of predicted transcripts (UCSC human genome assembly hg19 and splice variant specific FASTA sequences). The genes listed are newly added to the panel published in (de Bitter et al., 2017).

Gene symbol	Gene name	RefSeq mRNA ID (hg19)
**ADPGK**	ADP-dependent glucokinase	NM_031284
**ALK**	ALK receptor tyrosine kinase	NM_004304
**AR**	Androgen receptor, full length	NM_000044
**AXL**	AXL receptor tyrosine kinase	NM_021913
**BRAF**	B-Raf proto-oncogene, serine/threonine kinase	NM_004333
**CAT**	Catalase	NM_001752
**CD274**	Programmed Cell Death 1 Ligand 1	NM_014143
**CTLA4**	Cytotoxic T-Lymphocyte Associated Protein 4	NM_005214
**EGFR**	Epidermal growth factor receptor	NM_005228
**EGFRvIII**	Epidermal growth factor receptor variant III	FASTA
**ERBB2**	Human epidermal growth factor receptor 2 (HER2)	NM_004448
**ERBB3**	Human epidermal growth factor receptor 3 (HER3)	NM_001982
**ERBB4**	Human epidermal growth factor receptor 4 (HER4)	NM_005235
**FBP1**	Fructose-1,6-biphosphatase 1	NM_000507
**FGFR1**	Fibroblast growth factor receptor 1	NM_023110
**FGFR2**	Fibroblast growth factor receptor 2	NM_000141
**FOLH1**	Folate Hydrolase (Prostate-Specific Membrane Antigen, PSMA)	NM_004476
**IGF1R**	Insulin like growth factor 1 receptor	NM_000875
**VEGFR2 (KDR)**	Vascular endothelial growth factor receptor 2/Kinase insert domain receptor	NM_002253
**KIT**	KIT proto-oncogene receptor tyrosine kinase	NM_000222
**KLK3**	Kallikrein Related Peptidase 3 (Prostate-Specific Antigen, PSA)	NM_001648
**KRAS**	KRAS proto-oncogene, GTPase	NM_033360
**MERTK**	MER proto-oncogene, tyrosine kinase	NM_006343
**MET**	MET proto-oncogene, tyrosine kinase (variant 2)	FASTA
**METvar2d10**	MET variant 2, delta 10	FASTA
**METd4-5**	MET delta 4-5	FASTA
**METd7-8**	MET delta 7-8	FASTA
**METd14**	MET delta 14	FASTA
**MST1R**	Macrophage stimulating 1 receptor	NM_002447
**NTRK1**	Neurotrophic Receptor Tyrosine Kinase 1	NM_002529
**NTRK2**	Neurotrophic Receptor Tyrosine Kinase 2	NM_006180
**PDCD1**	Programmed cell death 1	NM_005018
**PDGFRA**	Platelet-derived growth factor receptor, alpha polypeptide	NM_006206
**PDGFRB**	Platelet-derived growth factor receptor, beta polypeptide	NM_002609
**PLXND1**	Plexin D1	NM_015103
**PTEN**	Phosphatase and tensin homolog	NM_000314
**RET**	RET proto-oncogene	NM_020975
**TP53**	Tumor protein p53	NM_000546

**VEGF (A)**	Vascular endothelial growth factor	FASTA
**VEGF121**	VEGF isoform 121	FASTA
**VEGF121b**	VEGF isoform 121b	FASTA
**VEGF165**	VEGF isoform 165	FASTA
**VEGF165b**	VEGF isoform 165b	FASTA
**VEGF189**	VEGF isoform 189	FASTA
**VEGF189b**	VEGF isoform 189b	FASTA
