# Supplementary material for: Molecular Profiling of Druggable Targets in Clear Cell Renal Cell Carcinoma Through Targeted RNA Sequencing
Source: Front Oncol. 2019 Mar 1;9:117. doi: 10.3389/fonc.2019.00117 (PMC6407434; doi:10.3389/fonc.2019.00117)
Supplement: Supplementary Table 2 — Gene expression in cluster a vs. cluster b (non-significant). Fold changes of cluster b/cluster a are indicated. Significance was determined using a Wilcoxon Mann-Whitney U-test with Benjamini Hochberg correction for multiple testing (p < 0.05, FDR < 0.01). Note that for significance, the p-value must not exceed the FDR. Significant differential gene expression in cluster a vs. cluster b is shown in Table 1. Fold changes are calculated as (cluster b/a), or -1/(cluster b/a) to prevent fold changes >0 and <1. FC, fold change. [file Table_2.pdf]

Supplementary Table SII. Gene expression in cluster a vs. cluster b (non-significant). Fold changes of cluster b/cluster a are indicated. Significance was determined using a Wilcoxon Mann-Whitney U test with Benjamini Hochberg correction for multiple testing ($p<0.05$, FDR<0.01). Note that for significance, the p-value must not exceed the FDR. Significant differential gene expression in cluster a vs. cluster b is shown in Table 1. Fold changes are calculated as (cluster b/a), or -1/(cluster b/a) to prevent fold changes >0 and <1. FC, fold change.

For gene expression levels in individual samples, see supplementary excel files.

Gene	Mean FPM Cluster a	Mean FPM Cluster b	P-value	FDR	FC Cluster b/a	Significant?
**FH**	433.10	182.34	0.004	0.003	-2.38	N
**IDH2**	1337.13	655.22	0.004	0.003	-2.04	N
**PGAM1**	482.42	810.02	0.004	0.003	1.68	N
**SDHC**	2775.33	1572.76	0.004	0.003	-1.76	N
**SLC16A7**	269.57	127.95	0.004	0.003	-2.11	N
**ACO2**	822.50	281.67	0.005	0.003	-2.92	N
**ATG4A**	153.21	93.77	0.005	0.003	-1.63	N
**CPT1A**	906.39	508.98	0.005	0.003	-1.78	N
**ENO1**	8921.33	17504.47	0.005	0.004	1.96	N
**GLUD1**	2424.01	1231.93	0.005	0.004	-1.97	N
**MDH1**	1035.21	476.61	0.005	0.004	-2.17	N
**VEGF**	130.47	649.38	0.005	0.004	4.98	N
**VEGF121**	253.32	1094.29	0.005	0.004	4.32	N
**CYCS**	799.54	413.20	0.008	0.004	-1.94	N
**IGF1R**	670.63	355.06	0.008	0.004	-1.89	N
**SDHB**	674.50	289.54	0.008	0.004	-2.33	N
**SDHD**	2089.99	912.76	0.008	0.004	-2.29	N
**SOD1**	1072.39	552.64	0.008	0.004	-1.94	N
**HK2**	13.79	105.38	0.011	0.004	7.64	N
**IDH1**	1478.08	616.60	0.011	0.004	-2.40	N
**IDH3B**	729.51	451.73	0.011	0.004	-1.61	N
**TP53I3**	255.24	139.34	0.011	0.004	-1.83	N
**TUBB**	1872.45	3205.56	0.011	0.004	1.71	N
**VEGF189**	538.16	2034.06	0.011	0.005	3.78	N
**VHLvar1**	204.43	113.02	0.011	0.005	-1.81	N
**ACLY**	1238.17	2239.27	0.015	0.005	1.81	N
**PGD**	632.18	385.22	0.015	0.005	-1.64	N
**PKM**	2049.59	5550.71	0.015	0.005	2.71	N
**PRKAA2**	221.09	150.08	0.015	0.005	-1.47	N

**KLK3**	4.56	0.83	0.017	0.005	-5.51	N
**FGFR2**	180.65	90.97	0.019	0.005	-1.99	N
**GLUL**	455.17	939.55	0.033	0.005	2.06	N
**IDH3G**	505.66	346.38	0.033	0.005	-1.46	N
**PRDX1**	1230.29	852.48	0.033	0.005	-1.44	N
**SDHA**	1986.26	1377.95	0.033	0.005	-1.44	N
**SOD2**	9163.55	17941.38	0.033	0.005	1.96	N
**PFKFB1**	8.07	0.78	0.035	0.005	-10.38	N
**AXL**	218.97	478.62	0.042	0.005	2.19	N
**BCAT1**	120.66	313.59	0.042	0.005	2.60	N
**PGK1**	2722.60	4627.50	0.042	0.006	1.70	N
**ACACA**	123.02	83.43	0.053	0.006	-1.47	N
**D2HGDH**	388.76	146.49	0.053	0.006	-2.65	N
**GPT**	143.71	20.65	0.053	0.006	-6.96	N
**HK3**	3.31	10.86	0.053	0.006	3.28	N
**NAPRT1**	71.62	20.78	0.053	0.006	-3.45	N
**VHLvar2**	141.77	70.81	0.053	0.006	-2.00	N
**CHKA**	99.87	62.60	0.066	0.006	-1.60	N
**PTEN**	1115.14	794.30	0.066	0.006	-1.40	N
**EGFR**	55.85	161.09	0.081	0.006	2.88	N
**NQO1**	598.09	248.85	0.081	0.006	-2.40	N
**PLXND1**	204.74	394.56	0.081	0.006	1.93	N
**FOLH1**	1241.49	747.21	0.119	0.006	-1.66	N
**METvar2d10**	12.51	26.47	0.119	0.006	2.12	N
**CBR1**	235.53	116.17	0.142	0.006	-2.03	N
**TXN**	5316.66	3578.78	0.142	0.007	-1.49	N
**AR**	155.75	81.78	0.168	0.007	-1.90	N
**PRKAA1**	364.52	297.91	0.168	0.007	-1.22	N
**SLC7A1**	189.12	272.49	0.168	0.007	1.44	N
**PDCD1**	1.11	3.81	0.176	0.007	3.42	N
**BRAF**	294.71	249.98	0.197	0.007	-1.18	N
**GCLC**	368.37	303.04	0.197	0.007	-1.22	N
**GCLM**	153.07	197.06	0.197	0.007	1.29	N
**HIF1A**	1314.51	792.40	0.197	0.007	-1.66	N
**HK1**	758.76	1009.56	0.197	0.007	1.33	N
**NAMPT**	505.30	838.70	0.197	0.007	1.66	N
**SLC2A3**	1951.53	3590.18	0.197	0.007	1.84	N
**SLC9A1**	426.40	253.99	0.197	0.007	-1.68	N
**CTLA4**	33.83	101.18	0.230	0.007	2.99	N
**MYC**	1200.02	1014.68	0.230	0.007	-1.18	N
**TALDO1**	1564.43	1426.44	0.230	0.008	-1.10	N

**CD274**	32.65	47.39	0.266	0.008	1.45	N
**GAD1**	50.13	32.98	0.266	0.008	-1.52	N
**METd14**	0.13	0.60	0.278	0.008	4.64	N
**FGFR1**	317.29	215.07	0.306	0.008	-1.48	N
**GPI**	1149.93	1375.49	0.306	0.008	1.20	N
**METd7-8**	0.29	0.93	0.327	0.008	3.25	N
**CKB**	1222.20	528.16	0.349	0.008	-2.31	N
**FASN**	53.75	40.37	0.349	0.008	-1.33	N
**KRAS**	27.69	19.00	0.349	0.008	-1.46	N
**PDGFRB**	849.70	575.46	0.349	0.008	-1.48	N
**EGLN1**	262.23	298.60	0.395	0.008	1.14	N
**ALK**	1.06	2.16	0.407	0.008	2.04	N
**METd4-5**	0.00	0.27	0.451	0.008	1.00	N
**ACTB**	6323.07	6890.94	0.497	0.008	1.09	N
**ERBB3**	808.40	632.80	0.497	0.009	-1.28	N
**VEGFR2**	880.37	1439.45	0.497	0.009	1.64	N
**ADPGK**	393.13	388.85	0.553	0.009	-1.01	N
**ARHGAP26**	938.83	936.52	0.553	0.009	-1.00	N
**C12orf5**	58.20	71.97	0.553	0.009	1.24	N
**TP53**	54.38	62.88	0.553	0.009	1.16	N
**NOX1**	0.14	0.26	0.588	0.009	1.86	N
**BCAT2**	146.69	142.04	0.612	0.009	-1.03	N
**VEGF121b**	0.00	0.18	0.644	0.009	1.00	N
**CBS**	20.40	17.86	0.672	0.009	-1.14	N
**EPAS1**	2169.06	2001.14	0.672	0.009	-1.08	N
**VEGF165b**	1.12	15.11	0.718	0.009	13.49	N
**MERTK**	475.81	554.85	0.735	0.009	1.17	N
**RPIA**	155.72	105.84	0.735	0.009	-1.47	N
**NOX3**	0.14	0.15	0.747	0.009	1.03	N
**GFPT1**	1070.69	1026.17	0.800	0.010	-1.04	N
**SLC1A2**	4.29	5.20	0.800	0.010	1.21	N
**SLC5A1**	56.94	296.79	0.866	0.010	5.21	N
**G6PD**	258.37	266.70	0.933	0.010	1.03	N
**EGFRvIII**	0.72	1.37	1.000	0.010	1.92	N
**PARP1**	666.66	597.17	1.000	0.010	-1.12	N
**PGK2**	0.11	0.62	1.000	0.010	5.89	N
